# Strong, tough and mechanically self-recoverable poly(vinyl alcohol)/alginate dual-physical double-network hydrogels with large cross-link density contrast

**DOI:** 10.1039/c8ra01302k

**Published:** 2018-05-08

**Authors:** Xuefeng Li, Mengmeng Shu, Han Li, Xiang Gao, Shijun Long, Tao Hu, Chonggang Wu

**Affiliations:** Hubei Provincial Key Laboratory of Green Materials for Light Industry, Hubei University of Technology Wuhan Hubei 430068 China li_xf@mail.hbut.edu.cn cgwu@mail.hbut.edu.cn; Collaborative Innovation Centre of Green Light-weight Materials and Processing, Hubei University of Technology Wuhan Hubei 430068 China; School of Materials and Chemical Engineering, Hubei University of Technology Wuhan Hubei 430068 China

## Abstract

Strong and tough poly(vinyl alcohol) (PVA)/alginate hydrogen-bonded-ionic dual-physical double-network (DN) hydrogels have been successfully prepared by a facile route of a freeze–thaw (25–25–25 °C) cycle followed by concentrated (1.0 mol L^−1^ of) aqueous-Ca^2+^ immersion of PVA/Na alginate (SA) mixed aqueous solutions. It was found that, at mole ratios of the PVA- to SA repeat units of 20/1 to 80/1, the DN gels likely evolved a semi-interpenetrating polymer network (IPN) morphology of rigid alginate networks dispersed in while interlocking with ductile PVA network to accomplish DN synergy that gave their high strength and toughness, where the high alginate rigidity originated probably from its dense cross-link induced syneresis and dispersion along crosslink-defective voids to result in little internal stress concentration. Tentatively mechanistically, as the 20/1–80/1 DN gels were stretched steadily, their mechanical response was gradually differentiated into distinct synergistic states: the sparsely hydrogen-bonded PVA served as a ductile matrix to bear small fractions of the established stresses at its large elongations; whereas the densely ionically (*i.e.* Ca^2+^) cross-linked alginate functioned as a rigid skeleton to sustain the remaining larger stresses upon its smaller local strains. Promisingly, this ductile-rigid matrix-skeleton synergistic mechanism of semi-IPN morphology may be universally extended to all A/B DN hydrogels of large A–B rigidity (or cross-link density) contrast, whether the cross-link nature of network(s) A or B is covalent, ionic, hydrogen bonded or van der Waals interacted. The strong and tough DN gels also displayed satisfactory self-recovery of viscoelastic behaviour, in that their Young's modulus and dissipated energy in the uniaxial tensile mode and dynamic storage and loss moduli in the oscillatory shear mode all recovered significantly from non-linear viscoelastic regimes despite different degrees of failure to revert to (quasi)linear viscoelasticity.

## Abstract 2

Intermediate compositions enable synergised, strong-and-tough dual-physical double-network hydrogels of dispersion-induced rigid, ionic alginate-networks interlocking with global ductile, hydrogen-bonded poly(vinyl alcohol)-network.

## Graphical abstract

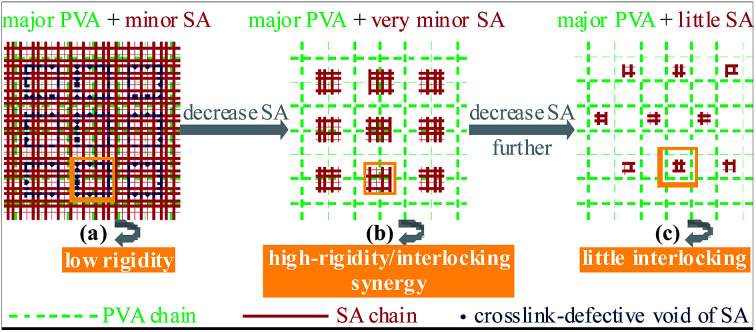

## Introduction

Owing to their superior properties such as excellent biocompatibility, quick multistimuli responses, low sliding friction and environmental friendliness, hydrogels have attracted increasing attention from researchers and found diverse applications in tissue scaffolds,^1,2^ drug release carriers,^3^ wound healing,^4,5^ artificial tissue replacements,^6,7^*etc.* However, as they are usually water-swollen polymer networks, hydrogels are mostly weak and/or brittle, which severely limits their biomedical applications. To overcome this disadvantage, many efforts have been focused on improving the mechanical properties of hydrogels to make them strong and/or tough, which include the strategic developments, over the last decade, of double-network (DN) hydrogels,^8–10^ nanocomposite hydrogels,^11,12^ fiber-reinforced hydrogels,^13^ microsphere-incorporated hydrogels^14^ and ionically cross-linked hydrogels.^15^

In these developments, double networking has been demonstrated to be an effective approach to enhancing the strength and/or toughness of hydrogels. As a typical representative of tough hydrogels, DN hydrogels feature a special dual-network structure of two types of polymeric components, which have opposite yet complementary mechanical responses to each other: usually, the minor strengthening component is a densely cross-linked, rigid skeleton serving as sacrificial bonds^16^ during an extensional process to sustain large stresses upon their small local strains, whereas the major elongational component constitutes a more sparsely cross-linked, ductile matrix acting as hidden chain lengths to bear the remaining smaller stresses at their larger stretches. In early years, conventional, chemically cross-linked (*i.e.* dual-covalent) DN hydrogels of high tensile strength (≥1.0 MPa) are synthesised generally *via* a two-step process of sequential free-radical polymerisations, in which a neutral, second network is polymerised within a preformed, first polymer-network swollen by an aqueous solution of the monomer and cross-linker for the polymerisation.^9^ However, the biological compatibility and thus applicability of these DN hydrogels may be largely problematic due to their completely synthetic and covalent nature.

People are later directed to incorporate (*i.e.* substitute) a physically cross-linked network due to the extraordinary mechanical properties (*e.g.* large elongation, high fracture- or tearing energy, low friction, *etc.*) of the resultant hybrid DN hydrogels. For instance, Sun *et al.*^17^ and Li *et al.*,^18,19^ respectively, used physical (essentially ionic) bonds of Ca carboxylate to replace the sacrificial, covalent bonds in the minor network skeleton, to form alginate/polyacrylamide (PAAm) ionic–covalent hybrid DN gels: despite their high water contents of more than 90 wt%, the gels are able to be stretched beyond 20 times their initial lengths and have fracture energies of up to ∼9000 J m^−2^, showing super-high stretchability and toughness;^17,18^ further, they exhibit super-lubrication behaviour in particular environmental solutions especially *aqua pura*.^19^ Yang *et al.*^20^ substituted multivalent cations (Al^3+^ or Fe^3+^) for divalent ones (Ca^2+^, Sr^2+^ or Ba^2+^) as the ionic cross-linker to strengthen alginate/PAAm hybrid gels, whose stretches are also observed to be above 13 upon the reinforcement. Combining a first, physical (essentially hydrogen-bonded) network of agar with a second, covalent network of PAAm, Chen *et al.*^21^ synthesised tough, hybrid DN hydrogels as well, which achieve high tearing energies of 500–1000 J m^−2^. Nevertheless, the hybrid DN gels usually have relatively weak tensile strengths (<0.30 MPa),^17–19,21^ maximally up to 1.0 MPa,^20^ in which the biocompatibility and toxicity issue of the covalent (*e.g.* PAAm) network also remains to be addressed.

Concomitant with the development of hybrid DN gels has been an emergence of dually physically cross-linked DN hydrogels in the past couple of years, which are found to possess tough yet weak mechanical properties as well. For example, based on their previous work,^21^ Chen *et al.*^22^ introduced a second, ductile network of PAAm copolymerised with hydrophobic stearyl methacrylate whose aggregates, mediated by the sodium dodecylsulfate surfactant, function as physical cross-links, to form agar/PAAm hydrogen bonded-van der Waals-interacted (dual-)physical DN gels by a one-pot method. Like the hybrid DN gels, these physical DN gels, although displaying high toughnesses (*e.g.* tearing energies of up to ∼1000 J m^−2^), are weak in tensile strength (<0.30 MPa);^22^ moreover, since their synthesis is a complicated process of the coupling of emulsification and free-radical copolymerisation, the residual, unreacted species present, especially the lipids (comonomer and surfactant molecules, micelles, *etc.*), may cause toxicity and/or incompatibility during bioapplications of the gels.

Exceptionally, Sun *et al.*^23^ used a binary polyampholyte of varying ion contents to form tough, highly-viscoelastic ionic single-network (SN) hydrogels. Although these physical gels have significantly high tensile strengths (usually up to ∼4 MPa) as well as very large stretches of up to ∼15, with the resultant ultrahigh tearing energies of maximally above 7000 J m^−2^, the polyampholyte networks, especially those of high ion contents, are subject to partial phase separation from the water medium (*i.e.* syneresis) during the gels preparation or application. This, probably a cause for the apparently high strengths, leads more or less to the low water contents (down to 60%), compositional heterogeneity and thus surface wrinkling (or corrugation) of the gels, which obviously compromise their applicability (*e.g.* safety, reliability, versatility, *etc.*) to biomedical applications; in addition, the synthetic polyampholytes may be still biologically incompatible to different extents.

In this work we present for the first time, to our knowledge, a facile and green approach to strong, tough and fully-biocompatible physical hydrogels, which are poly(vinyl alcohol) (PVA)/alginate sequential, hydrogen-bonded-ionic DN gels; further, their compositional dependencies of mechanical (tensile and tearing) properties are probed, based on which the mechanical self-recovery of the composition of gel having the maximum mechanical properties is identified. The stable, reproducible gel preparation process simply involves a freeze–thaw cycle of solution mixtures of PVA and alginate, both as purchased, to form the first PVA network followed by their immersion in a concentrated CaCl_2_ solution to construct the second alginate network, without any polymerisation composition required of monomer, comonomer (or cross-linker), initiator, surfactant, *etc.* More importantly, as specified by the U.S. Food and Drug Administration (FDA), both PVA and alginate are entirely applicable, safe biomedical materials.

## Experimental

### Materials

For the preparation of PVA/alginate DN gels as well as PVA- and alginate SN gels, PVA, sodium alginate (SA) (chemically pure) and CaCl_2_, anhydrous (analytical reagent, ≥96.0%) as an ionic cross-linker of the SA were all purchased from Sinopharm (Shanghai) Chemical Reagents Co., Ltd., China (Sinopharm), where the degrees of polymerisation and hydrolysis of the PVA are 1750 ± 50 and ≥99.0%, respectively. Prior to the gelations, deionised water, used for the preparation of all the aqueous solutions, was home made in our laboratory using a precision analytical ultra *aqua pura* machine (Pinguan, China, PGJ-10-AS).

Additionally, for the pregelation of the alginate SN gel, an ethylenediamine tetraacetic acid, Ca salt (EDTA–Ca) aqueous solution as a preliminary ionic cross-linker of the SA was prepared in our laboratory by mixing, in deionised water, CaCO_3_ (analytical reagent, ≥99.0%) with EDTA, disodium salt (EDTA–2Na) (dihydrate, ≥99.0%), both of which were purchased from Sinopharm, and d-(+)-glucono-δ-lactone (GDL) (≥99.0%), used as a controlled release agent for the Ca^2+^ of the aqueous EDTA–Ca, was supplied by Sinopharm as well.

All the chemicals purchased and made above, respectively, were used as received and prepared without any further purification.

### Preparation of PVA/alginate dual-physical DN hydrogels

The PVA and SA, respectively, were first stirred intensively with predetermined amounts of deionised water to both dissolve, at room temperature (RT) (∼25 °C), to make 25 wt% and 5 wt% of solutions. The two solutions were then mixed together vigorously, at RT for ∼4 h, at such different mass ratios that the mole ratios of PVA- to SA repeat units of the solution mixtures ranged from 20/1 to 80/1 (specifically 20/1, 30/1, 40/1, 50/1, 60/1, 70/1 and 80/1), under the assumption that the degrees of hydrolysis for PVA and of neutralisation for SA were both 100%. Upon deaeration *in vacuo* at RT for 20–30 min, the PVA/SA solution mixtures were subsequently poured into glass molds cavities measuring 100 × 100 × 3 mm^3^, and finally gelated in two steps as follows. First, the solution mixtures (contained in the molds) were subjected to a freeze–thaw (RT–−25 °C–RT) cycle of being frozen at −25 °C for 20 h and then thawed back at RT for at least 4 h to form a hydrogen-bonded network of the major PVA. Second, the preformed hydrogels each were taken out of the mold and immersed into a large amount of fresh CaCl_2_ aqueous solution of 1.0 mol L^−1^ at RT for at least 8 h, to ensure full ionic cross-linking of the SA *via* formation of Ca-dicarboxylate chelate triplets as well as complete swelling of the DN gel with water. In the first step, although the PVA crystallites formed during the freezing were largely responsible for the gelation,^24^ it was in essence the intersegmental hydrogen bonding of the low-crystallinity PVA that essentially facilitated its solution crystallisation and then stabilised the crystals against dissolution. As such, the PVA cross-linked morphology of crystallites accompanied possibly by non-crystalline hydrogen bonds is henceforward referred to typically as a hydrogen-bonded network of the PVA. During the second step, the immersion time of 8 h was found by gel weight monitoring to approximate the diffusion equilibria with respect to both Na^+^–Ca^2+^ ion exchange and water absorption–desorption for all the compositions of PVA/alginate gels prepared.

For comparison, PVA- and alginate SN hydrogels were also prepared. Similarly, the PVA SN gel was formed *via* subjection of a molded, deaerated PVA aqueous solution of ∼17 wt% (*i.e.* mean concentration of the PVA relative to water over the different compositions of PVA/SA solution mixtures)^25^ to the freeze–thaw cycle, followed by RT immersion of the off-mold gel into a large amount of fresh CaCl_2_ aqueous solution of 1.0 mol L^−1^ for at least 8 h. To mold a homogeneous square sheet, the alginate SN gel, however, had to be made differently than did the alginate network of the DN gels, using a stepwise (two-step) gelation method. First, a well-stirred, mixed aqueous solution of ∼2 wt% (*i.e.* mean concentration over the different compositions of PVA/SA solution mixtures)^25^ of SA, ∼2.2 × 10^−3^ mol L^−1^ of EDTA–Ca and ∼4.4 × 10^−3^ mol L^−1^ of GDL, all with respect to the contained water, was deaerated *in vacuo* and then decanted into a glass mold that subsequently stood at RT for 24 h, during which the Ca^2+^ ions of the EDTA–Ca were gradually released and exchanged into the SA to form Ca-dicarboxylate chelate cross-links, at a slow rate controlled by the GDL present. Second, the pregelated SA, contained in the mold, was then immersed into a large amount of fresh CaCl_2_ aqueous solution of 1.0 mol L^−1^ at RT for at least 8 h to ionically cross-link the SA further to completion.

Upon the preparation, each of the PVA SN, alginate SN and PVA/alginate DN hydrogels (referred to as PVA, alginate, PVA/alginate 20/1, 30/1, 40/1, 50/1, 60/1, 70/1 and 80/1, respectively) remained immersed in the same CaCl_2_ solution for RT storage to prevent its water content evaporation.

### Mechanical and rheological measurements

Each of the gels (square sheets), after taken out of the CaCl_2_ solution, was immediately cut into 50 mm (length) × 5 mm (width) rectangular-shaped specimens for tensile testing as well as for tensile hysteresis and self-recovery measurements, into 40 mm (length) × 10 mm (width) pants-shaped specimens with a longitudinally centred, curved V-shaped notch of 20 mm (length) × 5 mm (width) for tearing testing, and into discoid specimens of 25 mm in diameter for rotational rheometry, all of which were subsequently immersed back into the CaCl_2_ solution at RT for at least 1 day to relax any possible cutting-incurred internal stresses prior to the measurements. Immediately before the runs, the initial, precise thicknesses (mm) of all the specimens taken out of the CaCl_2_ solutions, fluctuating about the nominal thickness of 3 mm preformed from the glass molds, were measured using a micrometre for the tensile, tensile hysteresis, tensile self-recovery, tearing and rheological tests.

Tensile properties of the prepared hydrogels were evaluated at RT with a universal testing machine (MTS Systems (China) Co., Ltd., CMT4000) in the uniaxial tensile mode in the longitudinal direction of the specimens, at a grip distance of 25 mm and a crosshead speed of 50 mm min^−1^, using a 500 N load cell. During the testing, a stretch (mm mm^−1^), *ε*, was defined as the ratio of an elongated length (mm) to the initial length (mm) (*i.e.* grip distance of 25 mm) of the specimens, and the corresponding stress (MPa), *σ*, as the load (N) sensed from the load cell upon the stretch divided by the initial cross-sectional area (mm^2^) of 5 mm (width) × thickness (mm) of the specimens. In the resultant stress–stretch curves, the Young's modulus (kPa), *E*, was identified as the slope at the onset point of stretches (at or near the origin) where the stress began to become non-zero, the tensile strength (MPa), *σ*_max_, and elongation at break (mm mm^−1^), *ε*_max_, respectively, were defined as the stress and stretch at the fracture of the specimens, and the toughness (kJ m^−3^), *T*_s_, was denoted by the tensile work done until fracture per unit volume of the specimens, estimated as the integrated area underneath the curves. To ensure statistical significance of the results, each of the gels was tested with at least three parallel specimens, from which the stress–stretch curve corresponding to a median tensile strength was taken as the data for analysis.

Tearing tests of the gels were performed at RT with the same universal testing machine as the tensile tests but using a 100 N load cell. The grip distance between the two pants legs of the specimens, symmetrical with respect to the notch in the longitudinal direction, was set at 20 mm. One of the legs was then moved at a crosshead speed of 50 mm min^−1^ in the longitudinal direction to tear the specimens until fracture, during which the load (N), *F*, was recorded as a function of the displacement (mm), *x*. The tearing energy (J m^−2^), *T*_y_, as reported above in the literature^21–23^ as another measure of the gels toughnesses besides *T*_s_, was defined as the shear work required to tear up a unit area of the initial, half-notched cross section of 10 mm (width) × thickness (mm) of the specimens, as estimated by1
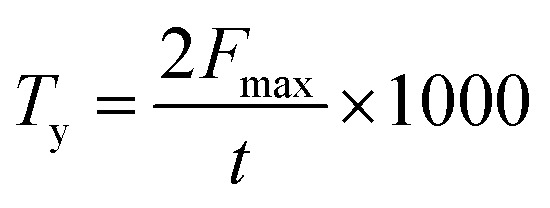
where *F*_max_ is the maximum load (N) read from the load–displacement curve across the tearing-to-fracture process, and *t* the initial thickness (mm) of the torn specimens. To minimise uncertainties of the results, a median *T*_y_ from the testing of at least three parallel specimens of each gel was regarded as its tearing energy for analysis.

After the tensile (*E*, *σ*_max_, *ε*_max_ and *T*_s_) and tearing (*T*_y_) tests of all the gels were completed, the composition (*i.e.* PVA/SA mole ratio) of PVA/alginate DN gel having maximum mechanical properties (*σ*_max_, *ε*_max_, *T*_s_ and *T*_y_) was identified, whose tensile hysteresis, tensile self-recovery and rotational rheology measurements were then carried out sequentially at RT. In the tensile hysteresis tests, a fresh specimen of the DN gel, standing gripped on the universal testing machine as it was over the whole process at an initial grip distance of 25 mm, was first elongated to a stretch and then unloaded to zero stretch both at a crosshead speed of 50 mm min^−1^; such a tensile-unloading cyclic test of the same specimen was repeated again and again without intermission across the cycles, sequentially to predetermined constant-incremental stretches to form a series of hysteresis loops. At its small stretches, a fresh specimen was tested from an initial stretch of 0.07 mm mm^−1^ until 0.55 mm mm^−1^ at an increment of 0.08 mm mm^−1^; while, at its larger stretches, another fresh specimen of the DN gel was measured from an initial stretch of 0.70 mm mm^−1^ until its fracture at an increment of 0.80 mm mm^−1^.

Tensile self-recovery, *i.e.* recovery behaviour of the tensile hysteresis, of the PVA/alginate DN gel of the maximum mechanical properties was tested as a function of time using the universal testing machine. For the runs, five fresh, parallel specimens of the DN gel, respectively, were first subjected to a tensile-unloading cycle described above of a predetermined stretch of 1.0 mm mm^−1^ to form their hysteresis loops; subsequently, they were all immersed into a large amount of fresh CaCl_2_ aqueous solution of 1.0 mol L^−1^ at RT, to prevent any changes in their water content and cross-link morphology, for different aging times of 0, 2, 10, 25 and 60 min, respectively; finally, they each were subjected to the same tensile-unloading cycle again to obtain the loops of the differently aged specimens. As the five fresh, parallel specimens had comparable hysteresis loop profiles to each other, only the hysteresis loop of the fresh specimen to be later aged for 0 min was selected as the one (*i.e.* control) for analysis. And, exceptionally, the hysteresis loop upon 0 min of aging of the control was realised by the tensile-unloading cycle performed *in situ* immediately after the control, with the specimen kept gripped as it was and actually not immersed into the CaCl_2_ solution for aging. All these data constituted the evolution (*i.e.* self-recovery) of the hysteresis loop with the aging time towards the control.

Rheology of the PVA/alginate gel of the maximum mechanical properties was measured on a rotational rheometer (TA Instruments, DHR-2) in the oscillatory shear mode with a parallel-plate fixture of 25 mm in diameter and 3 mm in separation at RT and a frequency of 1.0 Hz. First, a strain-amplitude sweep test, *i.e.* measurements of dynamic storage modulus (*G*′) and dynamic loss modulus (*G*′′) as functions of strain amplitude ranging from 0.1% to 1000%, of the gel was conducted with a fresh specimen to identify a critical strain amplitude above which its transition from a linear to non-linear viscoelastic behaviour, *i.e.* sharp changes in its constant (apparent) *G*′ and *G*′′, occurred. Then, an alternate-step strain-amplitude experiment of the gel was carried out without intermission for four cycles using another fresh specimen, between 0.1% (supposedly safely enough within its linear viscoelastic region) and one larger enough than the critical strain amplitude to fall well within its non-linear viscoelastic region, to investigate the fatigue of its dynamic shear self-recovery, *i.e.* of recovery behaviour of the hysteresis in response to applied oscillatory shear strains, from the non-linear towards linear viscoelastic regime.^26^ To run the experiment, in each of the cycles the specimen was first subjected to the linear strain-amplitude of 0.1% for ∼60 s immediately followed by the non-linear strain-amplitude for the same duration. In this context, both the *G*′ and *G*′′ of the gel were measured as functions of time until ∼480 s, for 10 times during every single semicycle of ∼60 s.

## Results and discussion

### Facile and green formation of PVA/alginate DN hydrogels

**Scheme 1:**
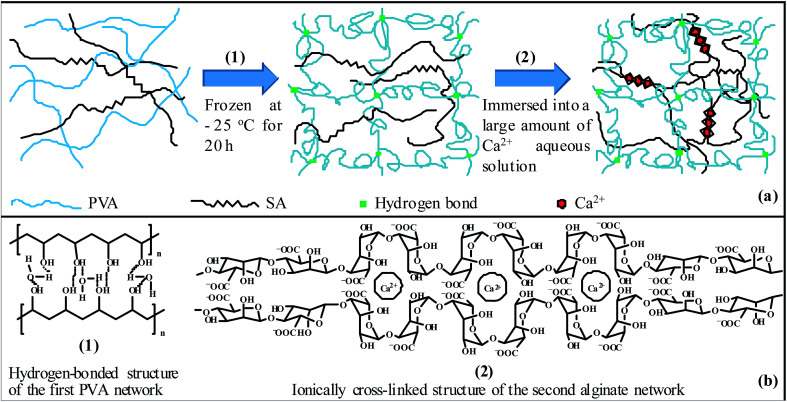
(a) Illustration of a sequential formation of poly(vinyl alcohol) (PVA)/alginate hydrogen-bonded-ionic dual-physical double-network (DN) hydrogels: (1) first, PVA/sodium-alginate (SA) aqueous solutions are subjected to a freeze–thaw (RT–−25 °C–RT) cycle of being frozen at −25 °C for 20 h and then thawed back at room temperature (RT) (∼25 °C) for at least 4 h to construct a hydrogen-bonded network of the major PVA; (2) second, the pregels are immersed into a large amount of fresh CaCl_2_ aqueous solution of 1.0 mol L^−1^ at RT for at least 8 h to establish an ionically cross-linked network of the minor SA through development of Ca-dicarboxylate chelate triplets. (b) Representation of the cross-link structures of the PVA/alginate DN hydrogels formed in (a): (1) the hydrogen-bonded structure of the first PVA network, showing a coexistence of mediate coordinations between the PVA hydroxyls that are bridged by a series of hydrogen-bonded water molecules and immediate interactions essentially present in the formed PVA crystallites; (2) the ionically cross-linked structure of the second alginate network.

In the PVA/SA aqueous solutions at RT, hydrogen bonds existed primarily between the PVA hydroxyls and their neighbouring water molecules as well as between the different neighbouring water molecules themselves, whereas there were little intermolecular hydrogen-bonded cross-links, either mediate or immediate, between the hydrated PVA chains. Once the solution mixtures were frozen at −25 °C for 20 h, a number of intermolecular hydrogen bonds were formed, mediately (primarily in non-crystalline regions) or immediately (mostly in crystallites), within the major PVA component to construct the first PVA network due to a much higher prevalence of hydrogen bonding at the considerably decreased temperature (50 °C lower than RT), while the SA macromolecules remained uncross-linked and homogeneously distributed in the PVA hydrogen-bonded network since intermolecular aggregate cross-links of Na-carboxylate ion pairs were impossible to develop within the minor SA component of so low concentrations (1–3 wt%^25^) in water and of such a high degree of Na^+^ neutralisation (supposedly nearly 100%) (*cf.*Scheme 1a(1)).^27^ The intermolecular hydrogen bonds in the PVA network might be immediate interactions, preferentially within the formed PVA crystallites, between the hydroxy groups of the PVA, yet could be also remote (*i.e.* mediate) coordinations between the PVA hydroxyls mediated (*i.e.* bridged) by a series of hydrogen-bonded water molecules under the intensively hydrated conditions (*cf.*Scheme 1b(1)).

Each of the so preformed PVA/SA gels, although subject to dissociation of part of the PVA intermolecular hydrogen bonds after thawed back at RT for at least 4 h, was still robust enough, due to the majority of the PVA component, to be subsequently immersed as a molded square sheet into a large amount of CaCl_2_ aqueous solution of 1.0 mol L^−1^ at RT for at least 8 h. During the immersion process, the Ca^2+^ ions were progressively diffused into the minor SA component to replace the Na^+^ ions out of the gels, establishing Ca-dicarboxylate ionic triplet cross-links largely intermolecularly to result in the second alginate network phase-separated from the first PVA network (*cf.*Scheme 1a(2)). The cross-linked structure of the alginate network is schematically represented in Scheme 1b(2), where, specifically, each Ca^2+^ ion was presumably coordinated to two bidentate carboxylates (*i.e.* their four O atoms) to form a chelate cross-link (not shown here). Concomitant with the immersion-induced Ca^2+^–Na^+^ ion exchange (*i.e.* SA gelation) was water absorption or desorption from or into the CaCl_2_ solution towards a thermodynamic equilibrium of the PVA/alginate two-phase hydrogel morphologies, during which the immersion time of 8 h was observed by gel weight monitoring to be adequate for realisation of the equilibria in both the cross-link density and water content of the two networks of the PVA/alginate DN gels investigated.

Similarly, the corresponding PVA- and alginate SN gels were prepared from respective PVA- and SA aqueous solutions of ∼17 and ∼2 wt%, which constituted the mean concentrations of the PVA and SA components relative to water, respectively, across the different compositions of PVA/SA mixed solutions (*cf.* the Experimental section). Although the concentration of the starting PVA solution might affect the hydrogen bond density of the freeze-thawed PVA network in either the SN or DN pregels, its subsequent immersion in the CaCl_2_ solution essentially offset this effect through water absorption or desorption giving rise to a degree of de-crosslinking or crosslinking, respectively, making the PVA SN gel have similar composition (*i.e.* water content) and morphology (primarily crosslink structure and -density) to those of each of the DN-gels PVA networks. By the same token, despite that it was prepared by the stepwise cross-linking of the ∼2 wt% of SA solution to ensure its composition homogeneity and surface smoothness, the alginate SN gel exhibited composition and morphology analogous to those of any of the DN-gels alginate networks formed by the single-step cross-linking of the 1–3 wt% of SA solutions, since it was the same Ca^2+^ immersion solution of 1.0 mol L^−1^ that concluded the ionic gelation processes of both.

In the preparation of the alginate SN gel and the DN-gels alginate networks, as the Ca^2+^ ions of as high as 1.0 mol L^−1^ were steadily exchanged *via* osmotic pressure into the dilute (1–3 wt%) of SA pregel and solutions, respectively, the ionic cross-link density of all the resulting alginate networks became exceptionally high due to the rich, strong electrostatic attractions to form Ca dicarboxylate triplets. This meanwhile induced significant synereses,^28^*i.e.* contractions of the alginate networks by their separation out of excess water, to dissimilar degrees leading to the much less hydrated alginate networks of rigidity having similar compositions and morphologies to each other, which was reflected evidently from a sharp volume shrinkage observed of the alginate SN gel upon completion of the Ca^2+^–Na^+^ exchange while inconspicuously from the DN gels owing to the very minority of their alginate networks. On the contrary, in the 13–20 wt% (ref. 25) of hydrated PVA networks of the SN and DN pregels (*i.e.* prior to the aqueous Ca^2+^ immersion), the hydrogen-bond cross-links formed upon the freeze–thaw cycle were rather limited in number density, since both the crystallites and the water-molecule bridges were largely subject to stochastic dissolution and disruption, respectively, due to the weak, labile nature of hydrogen bonding. Upon their subsequent immersion in the CaCl_2_ solution, the PVA networks of flexible chains were further swollen by water to dissimilar degrees as observed from the different volume increases of the PVA SN gel and the DN gels containing major PVA networks, which ultimately enhanced the hydrations and thus lowered the hydrogen-bond cross-link densities of the PVA networks both until similar levels approximating their equilibrium composition and morphology at RT, respectively.

Therefore, the PVA/alginate sequential, dual-physical DN hydrogels prepared in this work comprised a first, sparsely hydrogen-bonded, ductile and major network of PVA followed by a second, densely ionically cross-linked, rigid and minor network of alginate. Their formation, as presented above, was facile and green in virtue of the following three advantages. (1) It simply involved a freeze–thaw cycle followed by aqueous Ca^2+^ immersion of the PVA/SA mixed solutions. (2) As both the PVA and SA are commercially available, it did not require any (co)polymerisation from monomer, comonomer (cross-linker), initiator, surfactant, *etc.*, which obviously minimised the risk of biological incompatibility of the DN gels due to the absence of any residual suspicious species. (3) Most importantly, the PVA and alginate are both listed by the U.S. FDA as fully safe biopolymers, which further corroborated intrinsically the biological compatibility and safety of the PVA/alginate hydrogels prepared in our laboratory.

### Strong and tough PVA/alginate DN hydrogels as opposed to weak and brittle PVA and alginate SN hydrogels

**Fig. 1:**
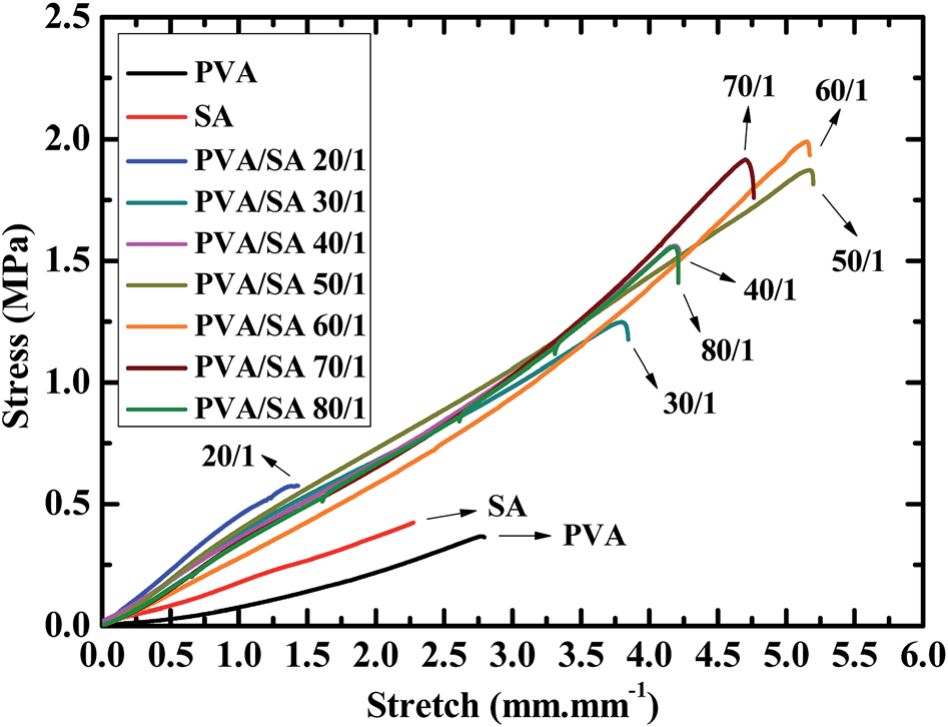
Stress, *σ vs.* stretch, *ε* curves, obtained by uniaxial tensile testing at room temperature (RT) (∼25 °C) at a crosshead speed of 50 mm min^−1^, of poly(vinyl alcohol) (PVA)/alginate sequential, hydrogen-bonded-ionic dual-physical double-network (DN) hydrogels having mole ratios of the PVA- to sodium alginate (SA) repeat units of 20/1, 30/1, 40/1, 50/1, 60/1, 70/1 and 80/1, compared with those of their corresponding PVA hydrogen-bonded and alginate ionic single-network (SN) hydrogels. Both the DN-gels PVA networks and the PVA SN gel are formed by hydrogen bonding of an ∼13–20 wt% of PVA aqueous solution upon its freeze–thaw (RT–−25 °C–RT) cycle of being frozen at −25 °C for 20 h and then thawed back at RT for at least 4 h, followed by its immersion into a large amount of Ca^2+^ aqueous solution of 1.0 mol L^−1^ at RT for at least 8 h, thus showing similar compositions (*i.e.* water contents) and morphologies (*i.e.* crosslink structures and -densities) to each other. Both the DN-gels alginate networks and the alginate SN gel are constructed through ionic cross-linking of an ∼1–3 wt% of SA aqueous solution with the large amount of Ca^2+^ immersion solution of 1.0 mol L^−1^ at RT for at least 8 h, hence displaying analogous compositions and morphologies to each other.

Fig. 1 shows the stress, *σ vs.* stretch, *ε* curves during the tensile testing of the PVA/alginate DN hydrogels having various mole ratios (20/1, 30/1, 40/1, 50/1, 60/1, 70/1 and 80/1) of the PVA- to SA repeat units as well as of their corresponding PVA- and alginate SN hydrogels, from which the *σ*_max_'s and *ε*_max_'s were read and the *E*'s and *T*_s_'s analysed of all the gels as given in Table 1. Also shown in Table 1 are all the *T*_y_'s obtained from the tearing tests. It is observed from Fig. 1 that, generally, although all the gels exhibited tensile quasilinear viscoelasticity, the PVA/alginate DN gels featured similar *σ*–*ε* behaviours to each other that were distinct from those of the PVA- and alginate SN gels: the DN gels looked mostly rigid, strong and tough (or ductile) whereas the SN gels appeared markedly softer, weaker and more brittle in that, qualitatively, the former basically displayed noticeably higher *E*, *σ*_max_, *ε*_max_ and *T*_s_ than the latter.

**Table 1:** Tensile properties (Young's moduli, *E*'s, tensile strengths, *σ*_max_'s, elongations at break, *ε*_max_'s, and toughnesses, *T*_s_'s) and tearing energies, *T*_y_'s, measured at room temperature (RT) (∼25 °C) at a crosshead speed of 50 mm min^−1^, of poly(vinyl alcohol) (PVA)/alginate sequential, hydrogen-bonded-ionic dual-physical double-network (DN) hydrogels having mole ratios of the PVA- to sodium alginate (SA) repeat units of 20/1, 30/1, 40/1, 50/1, 60/1, 70/1 and 80/1, against those of their corresponding PVA hydrogen-bonded and alginate ionic single-network (SN) hydrogels^a^

PVA/alginate hydrogel	*E* (kPa)	*σ*_max_ (MPa)	*ε*_max_ (mm mm^−1^)	*T*_s_ (kJ m^−3^)	*T*_y_ (J m^−2^)
PVA	49	0.36	2.8	408	63
Alginate	100	0.42	2.2	465	30
20/1	400	0.50	1.4	454	201
30/1	308	1.3	4.8	2494	402
40/1	270	1.5	4.2	3137	433
50/1	267	1.8	5.1	4875	446
60/1	257	1.9	5.1	4461	453
70/1	238	1.7	4.7	4071	441
80/1	220	1.5	4.2	3048	437

a Since they are both formed by hydrogen bonding of an ∼13–20 wt% of PVA aqueous solution upon its freeze–thaw (RT–−25 °C–RT) cycle of being frozen at −25 °C for 20 h and then thawed back at RT for at least 4 h, followed by its immersion into a large amount of Ca^2+^ aqueous solution of 1.0 mol L^−1^ at RT for at least 8 h, the DN-gels PVA networks and the PVA SN gel have similar compositions (*i.e.* water contents) and morphologies (*i.e.* crosslink structures and -densities) to each other. Likewise, as they are both constructed through ionic cross-linking of an ∼1–3 wt% of SA aqueous solution with the large amount of Ca^2+^ immersion solution of 1.0 mol L^−1^ at RT for at least 8 h, the DN-gels alginate networks and the alginate SN gel possess analogous compositions and morphologies to each other.

As more quantitatively presented in Table 1, the *E*, *σ*_max_, *ε*_max_, *T*_s_ and *T*_y_ of the SN gels, respectively, were only ∼50–100 kPa, ∼0.40 MPa, ∼2.5 mm mm^−1^, ∼450 kJ m^−3^ and ∼30–60 J m^−2^, with the *σ*_max_ and *T*_s_ of the alginate gel slightly higher than those of the PVA gel, the *ε*_max_ slightly smaller, however the *E* doubled (100 *vs.* ∼50 kPa) and the *T*_y_ halved (30 *vs.* ∼60 J m^−2^). This indicates that, although the SN gels exhibited comparably low tensile strengths and -toughnesses to each other, the alginate gel had a higher Young's modulus while a lower tearing toughness than the PVA gel despite their low *E*'s and *T*_y_'s, presumably associated with the much denser (ionic) cross-links of the former than the latter (*cf.* paragraph 4 of the last subsection).

Upon double networking of the PVA and alginate, the *E*, *σ*_max_, *ε*_max_, *T*_s_ and *T*_y_ of the DN gels (PVA/alginate 30/1, 40/1, 50/1, 60/1, 70/1 and 80/1) were significantly increased by 350–530% (or 120–210%), 220–380%, 70–110%, 450–1000% and 540–620% (or 1240–1410%), respectively, to 220–310 kPa, 1.3–1.9 MPa, 4.2–5.1 mm mm^−1^, 2500–4900 kJ m^−3^ and 400–450 J m^−2^ compared with those shown above of either of the SN gels (*cf.*Table 1), displaying strong synergy between the PVA and alginate networks in the overall mechanical properties. Exceptionally in the PVA/alginate (20/1) DN gel, although pronounced synergy occurred in the *E* (400 *vs.* ∼50–100 kPa) and *T*_y_ (200 *vs.* ∼30–60 J m^−2^) as well, little improvements were observed in the *σ*_max_ (0.50 *vs.* ∼0.40 MPa), *ε*_max_ (1.4 *vs.* ∼2.5 mm mm^−1^) and *T*_s_ (∼450 *vs.* ∼450 kJ m^−3^) on the double networking, suggesting that a minor alginate network of the largest 1/21 fraction of the DN gels investigated greatly compromised the tensile strength and -toughness of the DN gel well below those for the alginate fractions of 1/31 and smaller. Hence, it seems that the PVA/alginate mole ratio of 30/1 approximately constituted a critical ratio above which (*i.e.* at 30/1, 40/1, 50/1, 60/1, 70/1 and 80/1) the DN synergy arose significantly in the overall mechanical properties.

More delicately, the composition (*i.e.* PVA/alginate ratio) also had different effects on the *E*, *σ*_max_, *ε*_max_, *T*_s_ and *T*_y_ of the DN gels. As shown in Table 1, when the alginate content was reduced gradually (in the order the ratio was increased from 20/1 to 80/1), the *E* decreased monotonously whereas the *σ*_max_, *ε*_max_, *T*_s_ and *T*_y_ all initially increased, going through their respective maxima of 1.8–1.9 MPa, 5.1 mm mm^−1^, 4400–4900 kJ m^−3^ and 440–460 J m^−2^ invariably at an optimum ratio of 50/1–60/1, followed by decreases. Although the *E* decrease may be explained by a reduction in the volume fraction of the more densely cross-linked (*i.e.* higher *E*) alginate network with an increase in the PVA/alginate ratio, the peak-like changes of the *σ*_max_, *ε*_max_, *T*_s_ and *T*_y_ with the ratio obviously cannot be interpreted similarly by the volume (or mole) fraction effect. It is thus imperative that a distinct mechanism be proposed to address this issue, which has yet to be premised on the significant DN synergy in the above overall mechanical properties.

### A tentative ductile-rigid matrix-skeleton mechanism of the synergistic mechanical improvements in PVA/alginate DN hydrogels

As discussed in paragraph 3 of the first subsection, the PVA- and alginate networks, respectively, of each of the DN gels were comparable to the PVA- and alginate SN gels in terms of both composition (water content) and morphology (crosslink structure and -density). An issue is thus raised as to why the PVA/alginate hybrids at the various mole ratios investigated of 30/1 to 80/1 were all much more-rigid, stronger and tougher (or more stretchable) than their PVA- and alginate parents; and, further, why did their rigidity (*E*) decrease monotonically while strength and toughness (*σ*_max_, *ε*_max_, *T*_s_ and *T*_y_) show maxima with increasing the PVA/SA mole ratio of the hybrids from 20/1 to 80/1? To our understanding, these may be addressed by means of a tentative qualitative mechanism henceforth called the ductile-rigid matrix-skeleton model, with an emphasis on a crosslink-defective assumption of both the alginate SN gel and DN-gels alginate networks of similar compositions and morphologies to each other, as will be explicated in detail below.

In the PVA SN gel, the ready formation of hydrogen bond cross-links by the PVA crystallites as well as by the water-molecule bridges largely helped develop a homogeneous, continuous (*i.e.* uninterrupted) network of gelation, giving rise to no or little cross-link breakages at the length scale of a whole PVA macromolecular chain. Nevertheless, owing to the weakness and thus low number density of its hydrogen bond cross-links, the PVA SN gel, even with a nearly integrated network, had rather low *E*, *σ*_max_, *ε*_max_, *T*_s_ and *T*_y_ (*cf.*Table 1: ∼50 kPa, ∼0.40 MPa, ∼2.5 mm mm^−1^, ∼450 kJ m^−3^ and ∼60 J m^−2^, respectively) exhibiting mechanical softness, weakness and brittleness, as its failure occurred readily upon rupture of the intermolecular hydrogen bonding, secondary bonding forces, of a small concentration. In contrast, the alginate SN gel behaved differently. As discussed previously, the Ca-dicarboxylate ionic cross-links of the gel, which were difficult to be mediated by hydrogen-bonded water bridges due to their stronger electrostatic attractions, were primarily immediate and so densified upon immersion of the SA pregel in the concentrated Ca^2+^ solution of 1.0 mol L^−1^ that a significant syneresis was induced simultaneously to shrink the gel. Thus, it is not difficult to infer that the occurrence of syneresis, generally sequential in the gel thickness direction at the mercy of the Ca^2+^ diffusion, probably incurred fragmentation of the network into smaller subnetworks within the gel in the long run leaving lots of defects (*i.e.* interruptions) in between that were void of any ionic cross-links. Although the alginate gel's ionic cross-links were strong and dense, the rich defective voids among them functioning as stress concentration sites dictated its small stress (or load) responses to external stretches (or tears), resulting in its much lower *E*, *σ*_max_, *ε*_max_, *T*_s_ and *T*_y_ than if the alginate network would have been continuous without the inter-subnetwork cross-link interruptions, some of which (*σ*_max_, *ε*_max_ and *T*_s_) were comparable to those of the PVA gel (*cf.*Table 1).

**Scheme 2:**
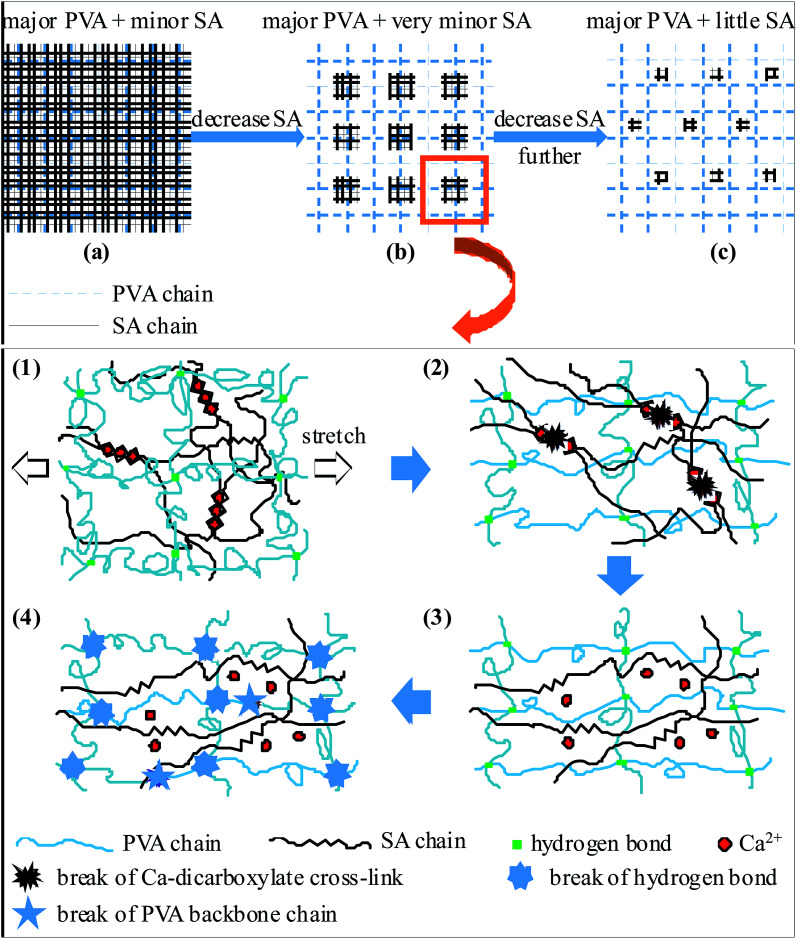
Representation of a tentative ductile-rigid matrix-skeleton mechanism of the synergistic mechanical enhancement in poly(vinyl alcohol) (PVA)/sodium alginate (SA) sequential, hydrogen bonded-ionic dual-physical double-network (DN) hydrogels: (a) the DN gel comprises a major PVA network sparsely hydrogen bonded and a minor SA network densely ionically cross-linked with Ca^2+^ to form an interpenetrating polymer network (IPN), which is mechanically dominated by the alginate network that, despite its high intrinsic rigidity, has as poor mechanical properties as its parallel alginate single-network (SN) gel due to the significant presence of internal crosslink-defective voids (*i.e.* stress concentration) upon a cross-link induced syneresis that poses its much lower effective rigidity; (b) with an increase in the PVA/SA ratio above a critical value, the DN gel turns into a semi-IPN of small, dispersed alginate networks locally interlocking with global PVA network, which enables synergy of the PVA-matrix's ductility and the alginate-skeleton's high effective rigidity from its little internal stress concentration upon crosslink-induced syneresis and dispersion along the crosslink-defective voids, and hence gives greater mechanical properties than either of the parallel PVA- and alginate SN gels; (c) with increasing the PVA/SA ratio further beyond another higher critical value, the DN gel evolves into an alginate-“filled” PVA where the smaller, dispersed alginate networks, although more rigid owing to the presence of fewer internal crosslink-defective voids, do not locally interlock and thus may have basically van der Waals interactions with the global PVA network, leading to a predominance of the weak PVA network that has comparably poor mechanical properties to its parallel PVA SN gel. Specifically, the PVA-alginate mechanical synergism in (b) during a tensile or tearing process is illustrated sequentially from (1) to (4): (1) the interlocking PVA and alginate networks deform synchronously and equally upon initial stretches of the DN gel; (2) the DN gel is progressively differentiated upon further stretches into a ductile PVA matrix and a rigid alginate skeleton of distinct mechanical responses (stretchable *vs.* stressable, respectively), until a large critical stretch of the former when the stress of the latter is large enough to begin to rupture the Ca dicarboxylate cross-links; (3) the sacrificial ionic bonds of Ca dicarboxylate are all broken upon a further small stretch from the onset of their rupture, leaving the hydrogen bond cross-links of the well-extended PVA network nearly intact; (4) the evolved large stress turns to be suffered essentially by the global, weak PVA network, resulting in a catastrophic failure of the DN gel upon instantaneous breakage of the intermolecular hydrogen bonds possibly accompanied by a small degree of backbone chain scission of the PVA.

When it comes to a hybridisation of the PVA- and alginate SN gels, things may change contingent upon the composition (*i.e.* PVA/SA mole ratio) of the resultant PVA/alginate DN gels. In the case the DN gel comprises such major PVA and minor alginate networks that an interpenetrating polymer network (IPN) is formed (Scheme 2a), the mechanical properties of the gel are dominated by the minor alginate network which has similar composition and morphology and thus comparably low *E*, *σ*_max_, *ε*_max_, *T*_s_ and *T*_y_ to the significantly crosslink-defective alginate SN gel. This is because, although both the networks are global, it is the much denser alginate network but not the sparse PVA network that primarily responds to external stretches (or tears) in the IPN. Once the alginate network is disrupted, the PVA network as well verges on fracture leading to failure of the DN gel, since the *σ*_max_'s of the two networks, approximating those of their respective SN-gel counterparts, are supposedly comparable to each other (*cf.*Table 1: 0.36 *vs.* 0.42 MPa).

When the PVA/SA mole ratio is increased beyond a critical value, the SA component is minor enough during its dense ionic cross-linking to fragment the formed network into so small ones, upon an intensive syneresis along crosslink-defective voids, that a number of local alginate networks, mostly free of internal cross-link defect any more, are dispersed in while interpenetrating with the major, global PVA network to form a semi-IPN (Scheme 2b). As the semi-IPN is initially stretched, small deformations of the PVA continuous network occur, which are transmitted synchronously and equally to the dispersed alginate networks interlocking with the PVA network (Scheme 2b(1)). With increasing the stretch of the DN gel steadily, its mechanical response is gradually differentiated into two distinct states: the PVA flexible chains, which are sparsely hydrogen-bonded globally, serve as a ductile matrix to bear only small fractions of the established stresses at its large elongations; meanwhile, the alginate chains in each of the local networks, zipped here and there by Ca^2+^ ions as densely as in the alginate SN gel, function as a rigid skeleton to sustain the remaining larger stresses upon its smaller local stretches. As the DN-gel's stretch reaches a certain level, the local stress within the alginate network domains is large enough to begin to unzip the chains (*i.e.* rupture the Ca dicarboxylate cross-links) (Scheme 2b(2)) until all such sacrificial ionic bonds are essentially broken, with the hydrogen bond cross-links of the well-extended PVA network nearly intact (Scheme 2b(3)). At this juncture, the developed large stress of the gel turns to be sensed primarily by the weak, global PVA network, resulting in its catastrophic failure presumably *via* instantaneous breakage of the intermolecular hydrogen bonds accompanied by a small degree of backbone chain scission of the PVA (Scheme 2b(4)).

When the PVA/SA mole ratio is further increased above another higher critical value, the dispersed defect-free alginate networks from a crosslink-induced syneresis are so smaller that nearly no interpenetration occurs between them and the PVA global network (Scheme 2c). Under this circumstance, there may be basically van der Waals forces (*e.g.* dipole–dipole interactions) between the PVA- and alginate networks that are not interlocking with each other, as if the highly cross-linked alginate domains simply functioned as a small amount of inert filler that obviously cannot play a role of rigid skeleton (or sacrificial bonds^16^) for effective mechanical reinforcement of the PVA network. This infers that the mechanical properties of the DN gel are essentially dictated by the weak PVA network which, as discussed previously, exhibits analogous composition and morphology and hence similarly low *E*, *σ*_max_, *ε*_max_, *T*_s_ and *T*_y_ to the PVA SN gel.

Therefore, it is summarised from the above discussion that the DN morphology of the major-PVA/minor-alginate gels, which changes with the PVA/SA mole ratio, greatly affects their mechanical properties. The PVA–alginate IPN (Scheme 2a) and alginate-“filled” PVA network (Scheme 2c) morphologies, respectively, are mechanically dominated by the crosslink-defective alginate network and the weak PVA network, both of which display as poor mechanical properties as their respective SN-gel counterparts. Nevertheless, the semi-IPN morphology (Scheme 2b), with the dispersed small alginate networks locally interpenetrating (*i.e.* interlocking) with the global PVA network, interestingly enables mechanical synergy between the PVA matrix and the alginate skeleton which, respectively, impart ductility (large stretches) and rigidity (large stresses) to the DN gel, leading to its higher *E*, *σ*_max_, *ε*_max_, *T*_s_ and *T*_y_ than either of the SN gels. In this ductile-rigid matrix-skeleton model (*cf.*Scheme 2b), the rationale behind the mechanical synergy lies in that (1) few cross-link defects occur inside the (very minor) skeleton upon a syneresis along the defective voids, such that its intrinsic rigidity is essentially retained without notable stress concentration, and that (2) the skeleton interlocks locally with the matrix, which ensures effective transmission and coordination of stretches and stresses and thus synergism of the matrix ductility and skeleton rigidity.

Returning to the different compositions of PVA/alginate DN gels prepared in this work, we observe that generally their mechanical properties (*E*, *σ*_max_, *ε*_max_, *T*_s_ and *T*_y_) were all enhanced significantly compared with the PVA- and alginate SN gels (*cf.*Table 1), which is likely due to the fact that the PVA/SA mole ratios used of 20/1 to 80/1 fell invariably within the compositional regime required for mechanical synergy of the semi-IPN morphology described in Scheme 2b. Hence, while a large improvement either in the strength (*σ*_max_) of dual-covalent DN gels^9^ or in the toughness (*ε*_max_, *T*_s_ or *T*_y_) of ionic-covalent,^17–20^ hydrogen bonded-covalent,^21^ hydrogen bonded-van der Waals-interacted,^22^*etc.* DN gels has been extensively reported against that of their corresponding SN gels, we have obtained for the first time in this work the green, biocompatible PVA/alginate hydrogen-bonded-ionic DN gels that are both significantly stronger (of larger *σ*_max_) and tougher (of higher *ε*_max_, *T*_s_ and *T*_y_) than the PVA- and alginate SN gels. This originates presumably from, to different extents, the aforementioned ductility–rigidity synergism exclusively in the DN gels upon stretch–stress differentiation, respectively, between the PVA matrix (*i.e.* global network) and the alginate skeleton (*i.e.* dispersed networks). However, particularly worth noting are the considerable increases in the gels *E*'s upon the double networking of PVA and alginate, which have been found as well in agar/PAAm hydrogen-bonded-covalent hybrid DN gels in both their tensile^29^ and compressive^30^ moduli; as the *E* of the PVA/alginate DN gels reflected, prior to the stretch–stress differentiation, the magnitude of their responding stress upon an initial, small shared stretch of the interlocking PVA- and alginate networks, this DN-gel rigidification arose obviously from a markedly enhanced rigidity of the minor alginate upon its syneresis and dispersion along the crosslink-defective voids (*i.e.* sharp reduction in stress concentration) before the PVA-alginate ductility–rigidity synergy stated above actually occurred.

Further, we may interpret the composition–property relationships, presented in Table 1, of the DN gels also in terms of the ductile-rigid matrix-skeleton model proposed based on Scheme 2b. In the semi-IPN morphology shown in Scheme 2b, there are primarily three factors that may affect the mechanical properties of the PVA/alginate DN gels, *i.e.* the volume fraction, rigidity and interlocking degree (with the PVA network) of the alginate networks, all of which change with the DN-gel composition (*i.e.* PVA/SA mole ratio). Since the alginate rigidity and interlocking degree were both high enough to have induced the pronounced DN mechanical synergy, the *E* of the DN gels, being their initial tensile response prior to an occurrence of the ductility–rigidity synergism, was probably dominated by the alginate volume fraction. This indicates that the *E*, rather high (∼400 kPa) at the PVA/SA mole ratio of 20/1, was reduced monotonously to ∼220 kPa with a steady increase in the PVA/SA ratio to 80/1 due essentially to a monotonic decrease in the volume fraction of the much more rigid alginate networks in the DN gels. Nevertheless, the strength and toughness (*σ*_max_, *ε*_max_, *T*_s_ and *T*_y_) of the DN gels obtained from the ductility–rigidity synergism were mechanistically much more sensitive to the immediate prerequisites (or rationale) for the synergism, *i.e.* alginate rigidity and interlocking degree, than to the alginate volume fraction. As the PVA/SA ratio was raised gradually from 20/1 to 80/1, a steadily increased SA minority (relative to the PVA) caused, upon a crosslink-induced syneresis, the formation of smaller alginate networks with fewer internal cross-link defects (*i.e.* higher rigidity) and meanwhile with a lower interlocking degree (*i.e.* less synergy) with the PVA network, which, respectively, contributed to an improvement and a deterioration in the mechanical properties (*σ*_max_, *ε*_max_, *T*_s_ and *T*_y_) of the DN gels. It is thus not difficult to infer that, when the PVA/SA ratio rose from 20/1 all the way to 80/1, the increased rigidity and decreased interlocking competed in such a fashion that first the effects of the former prevailed over those of the latter and then *vice versa*, giving rise to maxima in the DN-gel mechanical properties almost invariably at an optimum ratio of 50/1 to 60/1, as shown in Table 1 and discussed in the last subsection.

At this juncture, we take the liberty of summarising the general morphological requirement for mechanical synergy of A/B DN hydrogels of all the types (dual-covalent, covalent–physical hybrid and dual-physical), which may be a semi-IPN of local densely-crosslinked, rigid networks-A dispersed in and interlocking with global sparsely-crosslinked, ductile network-B as illustrated in Scheme 2b, whether the cross-link nature of network(s) A or B is covalent, ionic, hydrogen bonded or van der Waals interacted. Accordingly, the interpretive ductile-rigid matrix-skeleton model proposed in this work with an emphasis on the crosslink-defective assumption of network(s) A becomes universal as well, based on which the synergistically improved mechanical properties (basically modulus, strength and toughness) of the A/B DN gels may be tailored towards maxima by the following two consecutive approaches. (1) First, maximise the contrast between the cross-link densities and thus rigidities of networks A and B to accomplish both maxima in the intrinsic rigidity of networks-A skeleton and in the ductility of network-B matrix; (2) then, control the composition (*i.e.* A/B ratio) at such an optimal level that the minor networks-A obtained from a crosslink-induced syneresis and dispersion along the crosslink-defective voids are small enough to contain few cross-link defects (*i.e.* little stress concentration) internally to give high effective rigidity while, meantime, large enough to interlock fully with the network-B to ensure the DN synergy, thus making the combined mechanical effects maximised.

### Mechanical hysteresis and self-recovery of PVA/alginate DN hydrogels

**Fig. 2:**
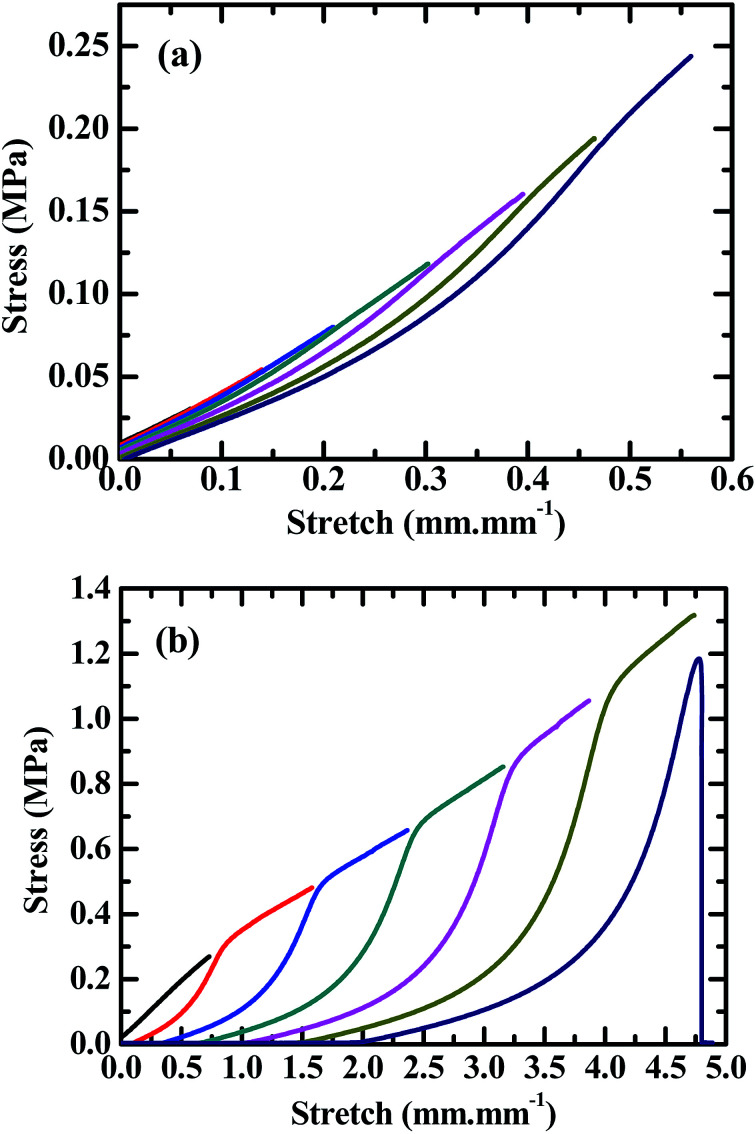
Tensile hysteresis loops, at room temperature (∼25 °C), of a poly(vinyl alcohol) (PVA)/alginate sequential, hydrogen-bonded-ionic dual-physical double-network (DN) hydrogel of maximum mechanical properties (tensile strength, elongation at break and toughness) having a mole ratio of the PVA- to Na alginate repeat units of 60/1, in which the Ca-dicarboxylate ionic cross-links of the minor, dispersed alginate networks are dramatically denser than the hydrogen bond cross-links of the major, global PVA network. To obtain the hysteresis loops, a fresh specimen of the DN gel, kept gripped on a universal testing machine as it is over the whole process at an initial separation of 25 mm, is subjected to sequential tensile-unloading (to zero stretch) cycles of predetermined constant-incremental stretches at a crosshead speed of 50 mm min^−1^ without intermission between the neighbouring cycles: (a) at its small stretches, one fresh specimen is run from an initial stretch of 0.07 mm mm^−1^ until 0.55 mm mm^−1^ at an increment of 0.08 mm mm^−1^; while (b) at its larger stretches, another fresh, parallel specimen is run from an initial stretch of 0.70 mm mm^−1^ until its fracture at an increment of 0.80 mm mm^−1^. As it essentially overlaps completely with the bottom of the immediately subsequent tensile curve, any of the unloading curves is omitted from either of Graphs a and b.

As discussed from all of the above, the PVA/alginate DN gels displayed the maximum mechanical properties (*i.e. σ*_max_, *ε*_max_, *T*_s_ and *T*_y_ of 1.8–1.9 MPa, 5.1 mm mm^−1^, 4400–4900 kJ m^−3^ and 440–460 J m^−2^, respectively) at an optimum composition (*i.e.* PVA/SA mole ratio) of 50/1 to 60/1; hence, the DN gel of 60/1 composition was selected to further investigate its tensile hysteresis and self-recovery behaviours in addition to the mechanical superiorities. Fig. 2 shows the hysteresis loops of the gel obtained from sequential tensile-unloading (to zero stretch) cycles of predetermined constant-incremental stretches, where Fig. 2a comprises a series of loops of a fresh specimen at its small stretches, from an initial stretch of 0.07 mm mm^−1^ until 0.55 mm mm^−1^ at an increment of 0.08 mm mm^−1^, while Fig. 2b includes a sequence of loops of another fresh, parallel specimen at its larger stretches, from an initial stretch of 0.70 mm mm^−1^ until its fracture at an increment of 0.80 mm mm^−1^. It should be stressed that, in either of Fig. 2a and b, a single fresh specimen of the gel, standing gripped on the universal testing machine as it was during the whole process, was subjected to its sequential tensile-unloading cycles one instantaneously after another without intermission between the neighbouring cycles. The unloading curve of a so formed hysteresis loop was reversible in that it generally overlapped fully with the bottom of the tensile curve of the immediately following loop, since both reflected essentially elastic stress–stretch behaviours of the same state, respectively, during the unloading and loading (*i.e.* tensile) processes of the same viscously deformed DN gel; therefore, to present the sequential hysteresis loops more clearly, all the unloading curves are omitted from both Fig. 2a and b. As discussed in the last subsection, the 60/1 PVA/alginate DN gel of the maximum *σ*_max_, *ε*_max_, *T*_s_ and *T*_y_ had an optimised semi-IPN morphology of highly rigid, dispersed alginate networks locally interlocking with highly ductile, global PVA network. During the gel's tensile process, the rigid alginate networks were such a densely ionically cross-linked skeleton that their blobs, *i.e.* chain segments between immediately neighbouring cross-links, had a rather small hydrodynamic volume and thus experienced small, primarily elastic stretches but giving rise to large stresses, whereas the blobs of the ductile, sparsely hydrogen-bonded PVA network matrix possessed a dramatically larger hydrodynamic volume, which therefore underwent significantly larger, essentially viscoelastic elongations yet leading to much smaller stresses. Particularly worth noting is that, according to the failure mechanism described in Scheme 2b(1)–(4), not until near the fracture of the DN gel did the ionic cross-links of the alginate networks and the hydrogen bonds of the PVA network begin to rupture sequentially. In this context, the viscoelastic behaviour of the gel shown in Fig. 2, regardless of its different levels of predetermined stretches, basically involved the viscous deformations only of the blobs between the stable cross-links of the PVA network that caused formation of the hysteresis loops, plus the elastic deformations both of the stable PVA- and alginate network blobs that dictated the unloading curve profiles of the hysteresis loops.

**Fig. 3:**
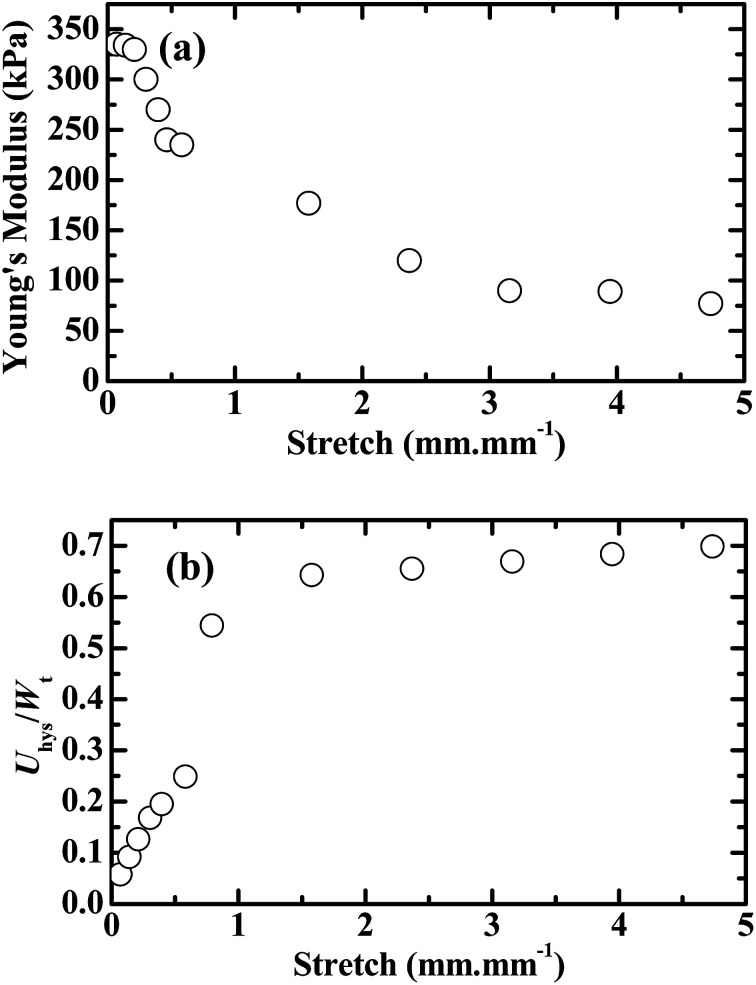
Effects of the (predetermined) stretch of a tensile-unloading (to zero stretch) cycle on (a) the Young's modulus (*E*_c_) of the cycle, *i.e.* slope at the onset point of the tensile curve of the cycle where the stress begins to become non-zero, and on (b) the degree of hysteresis (*U*_hys_/*W*_t_), *i.e.* ratio of the irreversible viscous work (*U*_hys_) to the total viscoelastic work (*W*_t_), until the cycle for a poly(vinyl alcohol) (PVA)/alginate sequential, hydrogen-bonded-ionic dual-physical double-network (DN) hydrogel of maximum mechanical properties (tensile strength, elongation at break and toughness) having a mole ratio of the PVA- to Na alginate repeat units of 60/1 and Ca-dicarboxylate ionic cross-links of the minor dispersed alginate networks dramatically denser than the hydrogen bond cross-links of the major global PVA network, which, standing gripped on a universal testing machine as it is across the entire process at an initial separation of 25 mm, is subjected to sequential tensile-unloading (to zero stretch) cycles of predetermined constant-incremental stretches at a crosshead speed of 50 mm min^−1^ without intermission between the neighbouring cycles: at its small stretches, one fresh specimen is run from an initial stretch of 0.07 mm mm^−1^ until 0.55 mm mm^−1^ at an increment of 0.08 mm mm^−1^; while, at its larger stretches, another fresh, parallel specimen is run from an initial stretch of 0.70 mm mm^−1^ until its fracture at an increment of 0.80 mm mm^−1^.

To quantitatively understand the viscoelastic behaviour of the 60/1 PVA/alginate DN gel reflected from its tensile hysteresis loops shown in Fig. 2 and 3 gives the dependences, on the (predetermined) stretch of a tensile-unloading (to zero stretch) cycle, of its Young's modulus, *E*_c_, of the cycle (Fig. 3a) and hysteresis degree, *D*_hys_, until the cycle (Fig. 3b), which were analysed from the Fig. 2 data as follows. The *E*_c_ of a cycle was read as the slope at the onset point of the tensile curve of the cycle where the stress began to become non-zero. The *D*_hys_ until a cycle, however, was evaluated by use of the following equation,2
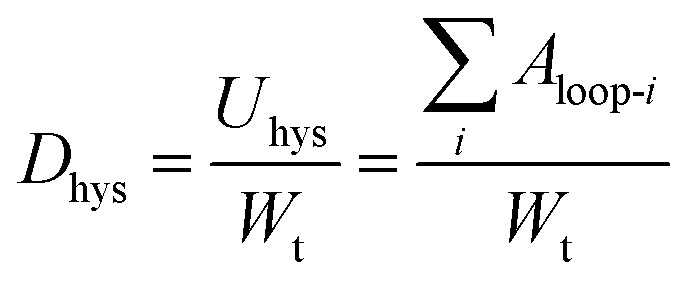
where *D*_hys_ is the degree of hysteresis until cycle *i* (*i* ≥ 1), *U*_hys_ the total dissipated energy from the hysteresis loops of cycles 1 − *i*, equivalent to the sum of the areas of loops 1 − *i* (*i.e.*
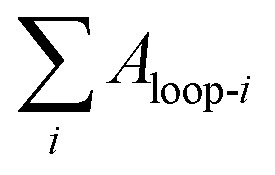
), and *W*_t_ the total tensile work done by cycles 1 − *i* (excluding the negative work by the unloading curve of cycle *i*), equal to the area integrated underneath the tensile quasilinear mastercurve comprising the consecutive tops of the tensile curves of cycles 1 − *i* (exceptionally, note that the entire tensile curve of cycle 1 constituted its top). It is seen from Fig. 3a that the *E*_c_ of the gel was decreased monotonously (from 335 to 77 kPa) with a steady increase in its (predetermined) stretch (from 0.07 to 4.70 mm mm^−1^), indicating that disentanglements of the PVA network blobs occurred progressively during their viscoelastic deformations at increasing length scales; the rationale behind this inference lies in that an increase in the chain entanglement points of a polymer usually enhances its modulus. Further shown in Fig. 3b is that the degree of hysteresis (*D*_hys_ or *U*_hys_/*W*_t_) of the gel, which represents the fraction of the irreversible viscous work (*U*_hys_) against the total viscoelastic work (*W*_t_) done to the gel, rose monotonically (from 0.06 to 0.70) as its stretch was raised persistently (from 0.07 to 4.70 mm mm^−1^). The fraction of the dissipated energy of 0.70 at the stretch of 4.70 mm mm^−1^ (near the fracture of the gel) is comparable to that (up to ∼0.67) of fractured vulcanised rubbers, whereas smaller than that (∼0.85) of a fractured poly(2-acrylamido-2-methylpropane sulfonic acid) (PAMPS)/PAAm dual-covalent DN hydrogel.^31^ This uncovers that, in terms of viscoelastic hysteresis, the mechanically synergistic PVA/alginate hydrogen bonded-ionic DN gel resembles a rubber covalent SN gel, both of which, however, are weaker than the PAMPS/PAAm dual-covalent DN gel due presumably to a PVA- or rubber SN of viscoelastic responses of the former against PAMPS/PAAm DNs of viscoelastic responses of the latter.

**Fig. 4:**
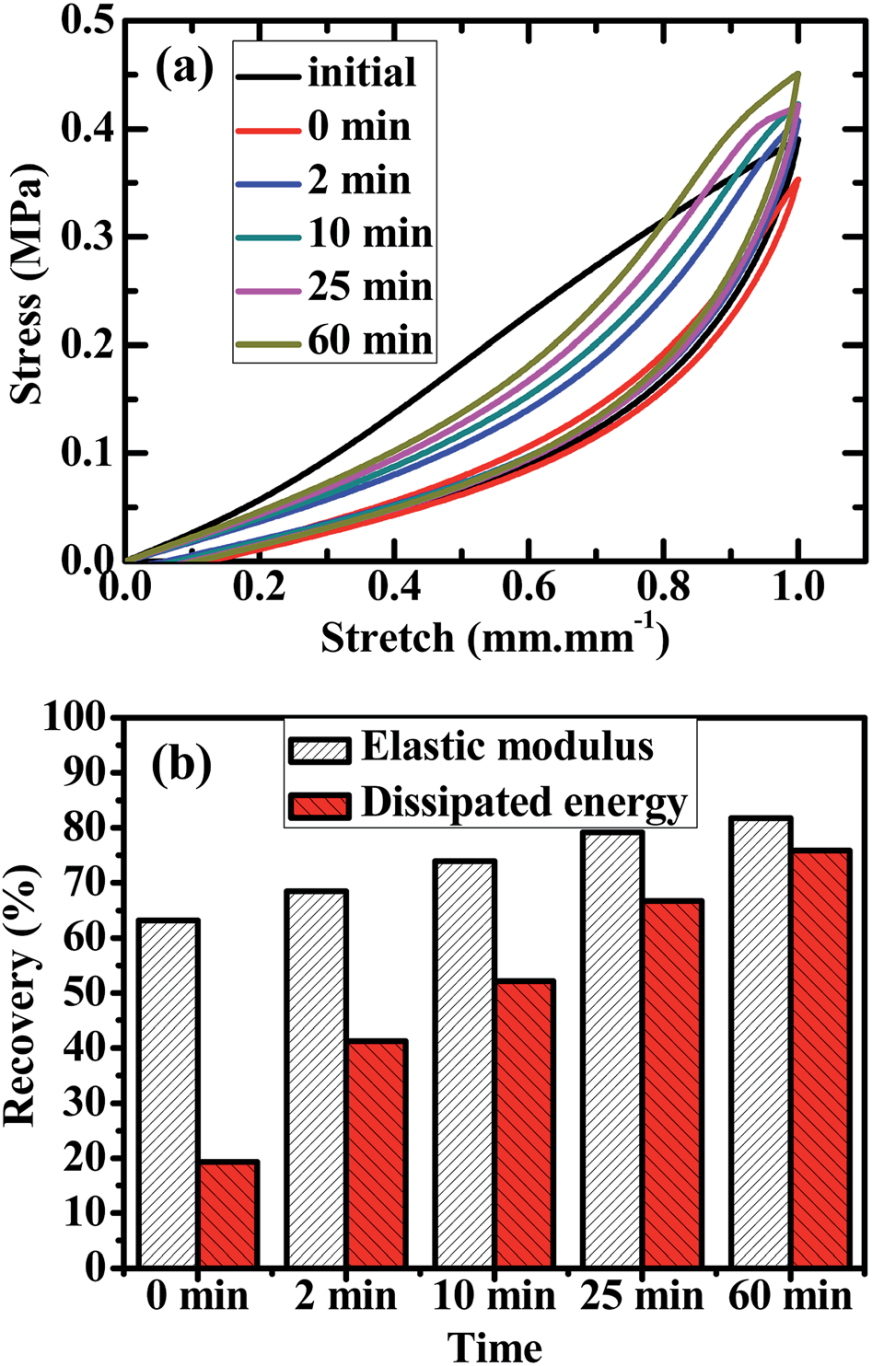
(a) Evolution, with the aging time upon a tensile-unloading (to zero stretch) cycle, of the hysteresis loop re-formed from the cycle for a poly(vinyl alcohol) (PVA)/alginate sequential, hydrogen-bonded-ionic dual-physical double-network (DN) hydrogel of maximum mechanical properties (tensile strength, elongation at break and toughness) having a mole ratio of the PVA- to Na alginate repeat units of 60/1 and Ca-dicarboxylate ionic cross-links of the minor dispersed alginate networks dramatically denser than the hydrogen bond cross-links of the major global PVA network. To conduct the experiments, five fresh parallel specimens of the gel first undergo a tensile-unloading cycle of 1.0 mm mm^−1^ stretch at a crosshead speed of 50 mm min^−1^ at room temperature (RT) (∼25 °C), then stand in a large amount of CaCl_2_ aqueous solution of 1.0 mol L^−1^ (*i.e.* age at the sacrifice of neither their water content nor cross-link morphology) at RT for increasing times of 0, 2, 10, 25 and 60 min, respectively, and are finally subjected to the tensile-unloading cycle again. Since the five fresh parallel specimens exhibit similar hysteresis loop profiles to each other, only the hysteresis loop of the fresh specimen to be subsequently aged for 0 min is shown as the initial one (*i.e.* control) for analysis; and, exceptionally, the hysteresis loop of the gel on 0 min of aging is realised by the tensile-unloading cycle run *in situ* immediately after the control, with the specimen kept gripped as it is and actually not immersed into the CaCl_2_ solution for aging. (b) Percent recoveries of the Young's (*i.e.* tensile elastic) modulus, *E*, and dissipated energy, *U*_hys_, towards those of the control as functions of the aging time for the PVA/alginate DN hydrogel of the maximum mechanical properties, where the *E* of all the loops (including the control) was read as the slope at the onset (*i.e.* non-zero stress) point of their tensile curve and the *U*_hys_ estimated as their area from (a).

Despite the tensile hysteresis discussed above of the 60/1 PVA/alginate DN gel, what concerns us more is the self-recovery of its hysteresis behaviour. To investigate this, hysteresis loops of the gel, formed from its tensile-unloading (to zero stretch) cycle of a predetermined stretch of 1.0 mm mm^−1^ at a crosshead speed of 50 mm min^−1^, were measured at the increasing aging times ranging from 0 to 60 min (specifically 0, 2, 10, 25 and 60 min) upon its fresh subjection to the same tensile-unloading cycle to form an initial loop (*i.e.* control), which is shown in Fig. 4a. More quantitatively, given in Fig. 4b are evolutions (*i.e.* percent recoveries), with the aging time, of the Young's (*i.e.* tensile elastic) modulus, *E*, and dissipated energy (*i.e.* irreversible viscous work done), *U*_hys_, of the hysteresis loops towards those of the control, where the *E* of all the loops (including the control) was read as the slope at the onset (*i.e.* non-zero stress) point of their tensile curve and the *U*_hys_ estimated as their area from Fig. 4a. It is observed that, on 0 min of aging, the hysteresis loop as a whole overlapped largely with the unloading curve of the control that was characteristic of the essentially elastic deformation recovery of the freshly viscoelasticity deformed (*i.e.* PVA-blob disentangled) gel (Fig. 4a), exhibiting both small *E* and *U*_hys_ recoveries, respectively, of 63% and 19% of the gel (Fig. 4b). As the aging time was steadily prolonged, the hysteresis loop became increasingly similar to the control both in area and locus (Fig. 4a), with *E* and *U*_hys_ recoveries of the gel gradually improving until 82% and 76%, respectively, up to 60 min of aging (Fig. 4b). This reveals that, apart from its maximum mechanical properties, the 60/1 PVA/alginate DN gel displayed satisfactory tensile self-recovery, *i.e.* recovery of tensile stress–stretch behaviour or of tensile hysteresis, after elongated up to a pre-failure stretch of 1.0 mm mm^−1^ that is practical for its bioapplications.

Nevertheless, the mechanism for the above tensile self-recovery is worthy of discussion. It is observed from Fig. 4 that, although the *E* and *U*_hys_ of the hysteresis loops of the gel recovered progressively to 82% and 76% those of its control, respectively, with increasing the aging time up to 60 min (Fig. 4b), the hysteresis loop at 60 min still differed essentially from the control (Fig. 4a). The unloading elastic curves of the loops, regardless of their aging times, all overlapped closely with that of the control (Fig. 4a), disclosing that the combined elastic deformation behaviours of the PVA- and alginate blobs of the differently aged gels were analogous to each other, and to that of the fresh gel as well, within the same stretch of 1.0 mm mm^−1^; however, although the loading (*i.e.* tensile) viscoelastic curve of the loops became increasingly crossed with that of the control with a gradual increase in the aging time, the aged gels essentially showed different tensile curve profiles from the fresh gel in that the former invariably exhibited a non-linear viscoelastic behaviour whereas the latter displayed a quasilinear viscoelastic behaviour (Fig. 4a). This contrast resolves that the viscous deformation effect of the PVA blobs of the aged gels, which increased with the aging time as reflected from the *U*_hys_ values, accounted primarily for their dissimilarity in stress–stretch viscoelastic behaviour from the fresh gel. In other words, even upon adequate (*e.g.* up to 60 min) of aging, the gel might still develop a PVA-blob entanglement morphology distinct in nature from that of the fresh one, which dictated their different viscous deformation behaviours from each other and hence the non-linear viscoelasticity of the former *vs.* the quasilinear viscoelasticity of the latter.

It is therefore surmised that, for the fresh PVA/alginate DN gel, the viscoelastic deformation of the PVA blobs in the tensile process progressively extended and disentangled their chain segments, which was followed by their complete elastic deformation recovery in the unloading process to form viscously deformed, re-coiled yet disentangled (*i.e.* entropy-decreased) PVA blobs. During the subsequent aging of the gel, the highly hydrated, stress-free PVA blob coils of disentanglement might be dominated by a Brownian motion (*i.e.* thermal diffusion or short-range viscous deformation) of their chain segments, which, thermodynamically, drove the PVA blobs to recover their conformation steadily towards one of increased entropy, *i.e.* to re-entangle themselves gradually, possibly at a smaller length scale than the entangled PVA blobs of the fresh gel. Thus, the re-entangled PVA blobs increased the *E*, their viscous deformation regenerated a *U*_hys_ while the re-entanglement in the new mode caused a different tensile curve profile of the gel during its final tensile-unloading cycle. This may tentatively explain why the aged gels, although their *E* and *U*_hys_ recovered stepwise to those of the fresh gel with increasing the aging time (Fig. 4b), exhibited the stress–stretch behaviour (non-linear) differing from that of the fresh gel (quasilinear) regardless of the aging time used (Fig. 4a).

**Fig. 5:**
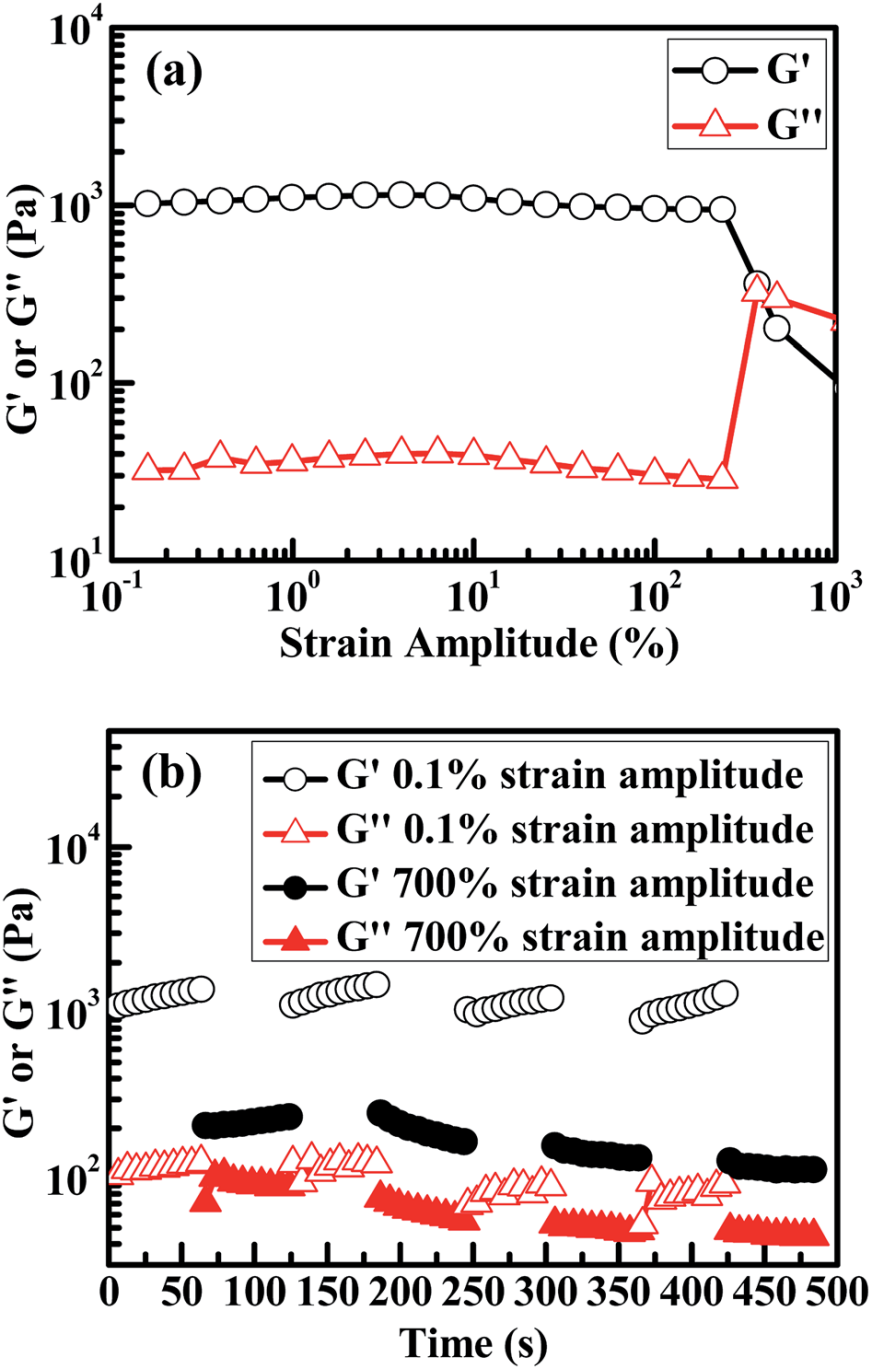
Rheology, in the oscillatory shear mode under a 25 mm parallel-plate fixture at room temperature (∼25 °C) and a frequency of 1.0 Hz, of a poly(vinyl alcohol) (PVA)/alginate sequential, hydrogen-bonded-ionic dual-physical double-network (DN) hydrogel of maximum mechanical properties (tensile strength, elongation at break and toughness) having a mole ratio of the PVA- to Na alginate repeat units of 60/1, in which the Ca-dicarboxylate ionic cross-links of the minor, dispersed alginate networks are dramatically denser than the hydrogen bond cross-links of the major, global PVA network: (a) plots of strain-amplitude sweep (*i.e.* step strain-amplitude) from 0.1% to 1000% using a fresh specimen, where a critical strain-amplitude beyond which the viscoelastic behaviour of the gel transitions from linear to non-linear is identified as ∼300%, as reflected from the onset point of sharp changes in its constant (apparent) dynamic storage and loss moduli, *G*′ and *G*′′ respectively; (b) plots of step strain-amplitude alternate without intermission between 0.1% and 700% for four cycles using another fresh parallel specimen, where the 0.1% is safely enough within the linear viscoelastic region of the gel as revealed from (a) while the 700% larger enough than the critical strain-amplitude, ∼300%, obtained from (a) to fall well within its non-linear viscoelastic region, thus showing the fatigue behaviour of the gel's dynamic shear self-recovery from the non-linear towards linear viscoelastic regime.

Discussed above has been recovery of the hysteresis (*i.e.* viscoelastic) behaviour of the 60/1 PVA/alginate DN gel from non-linear to (quasi) linear in the uniaxial tensile mode. By analogy, recovery of the gel's viscoelasticity from non-linear to linear was studied as well in the dynamic (*i.e.* oscillatory) shear fashion as presented in Fig. 5. At RT and a 1.0 Hz frequency, a strain-amplitude sweep test of the gel shown in Fig. 5a identified a critical strain-amplitude of ∼300%, above which its viscoelasticity transitioned from linear to non-linear in that the constant (apparent) dynamic storage and loss moduli (*G*′ and *G*′′, respectively) changed sharply. A subsequent alternating-step strain-amplitude measurement of the gel was conducted between 0.1% and 700% without intermission for four cycles as given in Fig. 5b, where the 0.1% and 700% fell well within its linear and non-linear viscoelastic regions, respectively, according to the critical 300% identified from Fig. 5a. It is seen that the non-linear *G*′ and *G*′′ @700% in the first cycle transitioned to the linear *G*′ and *G*′′ @0.1% in the second cycle whose magnitudes were comparable to those in the first cycle; this indicates that, unlike the aged tensile self-recovery described in Fig. 4, the non-linear viscoelasticity of the gel, even unaged, recovered almost completely towards its prior linear viscoelasticity in the oscillatory shear mode. Nevertheless, a further strain-amplitude alternation until the fourth cycle revealed that the non-linear to linear viscoelastic recovery of the gel fatigued with an increase in the alternation beyond the second cycle: while the *G*′ @0.1% changed little with the cycle, the *G*′′ @0.1% decreased considerably as from the third cycle, a combination of which suggests that the gel's viscoelasticity remained non-linear @0.1% in the third cycle despite a significant recovery from the non-linearity @700% in the second cycle; this behaviour was similar to the aged tensile recovery of the gel shown in Fig. 4.

## Conclusions

For the first time to our knowledge, green, strong and tough PVA/alginate dual-physical DN hydrogels have been successfully developed in this work by a facile approach of a freeze–thaw (25–−25–25 °C) cycle followed by concentrated (1.0 mol L^−1^ of) aqueous-Ca^2+^ immersion of PVA/SA mixed aqueous solutions, respectively, to form a sparsely hydrogen-bonded, global network of the major PVA followed by densely ionically (*i.e.* Ca^2+^) cross-linked, dispersed networks of the very minor alginate. The sequential DN gels are green in that their preparation does not involve a (co)polymerisation composition of monomer, comonomer (cross-linker), initiator, surfactant, *etc.* whose residues may compromise their biocompatibility, but, more importantly, starts directly from commercially available PVA and alginate that are both certified by the U.S. FDA as fully safe biomaterials.

Two factors, rigidity and interlocking degree (with the PVA network) of the alginate network(s), may primarily affect the strength and toughness of (major PVA)/(minor alginate) DN gels of a vast range of compositions (*i.e.* mole ratios of the PVA- to SA repeat units). At small PVA/SA mole ratios (*e.g.* <20/1), the alginate interlocking with PVA is large enough to form an IPN morphology of double global networks, when the DN gels are mechanically dominated by their denser alginate network; however, the Ca-dicarboxylate ionic cross-links are so densified that a significant syneresis occurs sequentially into the alginate network to fragment it, which leaves a lot of crosslink-defective voids (*i.e.* stress concentration sites) in between the resultant subnetworks that dictate its small effective rigidity, thus low strength and toughness. At large PVA/SA ratios (*e.g.* >80/1), a similar syneresis induces the extremely minor alginate network to fragment along crosslink-defective voids into dispersed ones which, although retaining their large intrinsic rigidity because of the presence of little stress concentration, are so small as to cause the DN gels to essentially form an alginate-“filled” PVA network morphology of little interlocking; in this case, the DN gels are mechanically dominated by their weak PVA network that has low strength and toughness. Only at intermediate PVA/SA ratios (*e.g.* 20/1–80/1 applied in this work) do the DN gels evolve a semi-IPN morphology of rigid alginate networks dispersed in while interlocking effectively with global PVA network, when mechanical synergy arises between the ductile PVA matrix and rigid alginate skeleton to give the DN gels high strength and toughness. More delicately across the compositions of 20/1 to 80/1, both the strength and toughness of the DN gels as well first improve and then deteriorate, giving rise to the highest (*σ*_max_ of ∼1.9 MPa; *ε*_max_, *T*_s_ and *T*_y_ of ∼5.1 mm mm^−1^, ∼4500 kJ m^−3^ and ∼450 J m^−2^, respectively) invariably at an optimum composition of ∼60/1, which indicates that the increased rigidity and decreased interlocking compete in such a fashion that the maximum synergy occurs at that composition.

Tentatively, the above ductile-rigid matrix-skeleton synergism is mechanistically described as follows: (1) initial small stretches of the semi-IPN are transmitted equally to its interlocking PVA network and alginate networks; (2) as the stretch is steadily increased, its mechanical response is gradually differentiated into distinct synergistic states—the sparsely hydrogen-bonded PVA serves as a ductile matrix to bear small fractions of the established stresses at its large elongations whereas the densely ionically cross-linked alginate functions as a rigid skeleton to sustain the remaining larger stresses upon its smaller local strains; (3) when the stretch reaches a certain level, the local stress within the alginate networks is large enough to rupture the Ca dicarboxylate cross-links until all of them are essentially broken, with the hydrogen bond cross-links of the well-extended PVA network nearly intact; (4) at this juncture, the evolved large stress of the semi-IPN turns to be sensed primarily by the weak PVA network, resulting in its catastrophic failure presumably *via* instantaneous breakage of the intermolecular hydrogen bonds accompanied by a small degree of backbone chain scission of the PVA. This synergistic mechanism of semi-IPN morphology may be universally applied to all the types of (*i.e.* dual-covalent, covalent–physical hybrid and dual-physical) A/B DN hydrogels of large A–B rigidity (or cross-link density) contrast for accomplishment of both their high strength and toughness, whether the cross-link nature of network(s) A or B is covalent, ionic, hydrogen bonded or van der Waals interacted.

As a typical representative of the mechanical synergy, the 60/1 PVA/alginate DN gel of the highest strength and toughness is found to exhibit satisfactory mechanical self-recovery. In the uniaxial tensile mode, the (quasi)linear viscoelastic hysteresis of the gel is augmented monotonously with a steady increase in the stretch until the elongation at break; at a 1.0 mm mm^−1^ stretch practical for the gel's bioapplications, its viscoelastic behaviour (or hysteresis) is considerably recoverable in that the Young's modulus and dissipated energy of the aged gels recover gradually towards those of the (fresh) gel by 82% and 76%, respectively, with increasing the aging time of the as-stretched gel of non-linear viscoelasticity up to 60 min despite their failure to revert to linear viscoelasticity. In the oscillatory shear mode, step strain-amplitude rheometry of the (fresh) gel alternating between its linear and non-linear viscoelasticity reveals that, although unaged, it initially displays a nearly complete non-linear to linear viscoelastic recovery, which then fatigues as the alternation proceeds since the later recoveries, although still significant, occur from one strong towards another remarkably weaker non-linear viscoelastic regime.

## Conflicts of interest

There are no conflicts of interest to declare.

## Supplementary Material
